# Proliferation-Diffusion Modeling in Glioblastoma: Impact of Supramaximal Resection on Survival

**DOI:** 10.3390/cancers17060995

**Published:** 2025-03-15

**Authors:** Maria Pia Tropeano, Zefferino Rossini, Ettore Bresciani, Andrea Franzini, Beatrice C. Bono, Pierina Navarria, Elena Clerici, Matteo Simonelli, Marta Scorsetti, Marco Riva, Letterio Salvatore Politi, Federico Pessina

**Affiliations:** 1 Department of Biomedical Sciences, Humanitas University, Via Rita Levi Montalcini 4, Pieve Emanuele, 20090 Milan, Italy; 2 Neurosurgery Department, IRCCS Humanitas Research Hospital, Via Manzoni 56, Rozzano, 20089 Milan, Italy; 3 Radiotherapy and Radiosurgery Department, IRCCS Humanitas Research Hospital, Via Manzoni 56, Rozzano, 20089 Milan, Italy; 4 Department of Medical Oncology and Hematology, Humanitas Clinical and Research Center—IRCCS, Humanitas Cancer Center, 20090 Milan, Italy; 5 Department of Neuroradiology, IRCCS Humanitas Research Hospital, Via Manzoni 56, Rozzano, 20089 Milan, Italy

**Keywords:** supramaximal resection, glioblastoma, proliferation, MGMT

## Abstract

Purpose: To evaluate the role of tumor invasiveness profile in a homogeneous cohort of patients with newly diagnosed GBM (2021 WHO) that underwent SUPR by the RANO criteria, and to analyze its impact on survival outcomes. Methods: Patients with newly diagnosed, histologically confirmed glial tumors featuring contrast-enhancing lesions, who underwent surgery at our institution between January 2007 and January 2024, were retrospectively reviewed. Preoperative total tumor volume (T-TV), contrast-enhancing (CE), and infiltrative FLAIR tumor volume (FLAIR-TV) were calculated in cubic centimeters (cc) via manual segmentation. A neuronavigation system was utilized for surgery and lesions were molecularly evaluated following the 2021 WHO CNS tumor classification. Therefore, all patients were classified into extent of resection categories by the 2022 RANO-Resect classification. The tumor invasiveness profile was assessed using the proliferation/diffusion (ρ/D) ratio, calculated following Swanson’s method. A statistical analysis was finally performed. Results: Between 2007 and 2024, 410 adult patients with newly diagnosed gliomas were treated at our institution. Methylation of the MGMT promoter was statistically significant (HR = 0.43, 95% CI: 0.20–0.94, *p* = 0.035), indicating that methylation has a protective effect on survival. In multivariate analysis, only MGMT status was confirmed to be an independent predictor of overall survival (OS). MGMT methylation was significantly associated with improved progression-free survival (PFS) in moderately diffuse tumors (HR = 0.18, 95% CI: 0.03–0.95, *p* = 0.044). Conclusions: Using the proliferation–diffusion model to classify tumors, we identified moderately diffuse tumors with methylated MGMT status as a subgroup with significant survival benefits from SUPR.

## Simple Summary

Glioblastoma (GBM) aggressive growth, early recurrence, and poor prognosis highlight the need for innovative therapeutic strategies. This single-center retrospective study is aimed to evaluate the role of the tumor invasiveness profile, assessed through the ρ/D ratio, in a homogeneous cohort of patients with newly diagnosed GBM-2021 WHO that underwent supramaximal resection (SUPR) according to RANO criteria, and to analyze its impact on survival outcomes. This is the first study that integrates the new RANO classification system for the extent of resection (EOR) in GBM surgery and correlates it with the tumor invasiveness profile. Using the proliferation–diffusion model to classify tumors, we identified moderately diffuse tumors with methylated MGMT status as a subgroup with significant survival benefits from SUPR.

## 1. Introduction

Despite advancements in surgical resection and intensive adjuvant therapies, glioblastoma (GBM) remains incurable, with no treatment achieving complete eradication or long-term control [1,2,3].

Although substantial progress has been made, critical questions about GBM remain unanswered. Its aggressive growth, early recurrence, and poor prognosis highlight the need for innovative therapeutic strategies.

Tumor malignancy is biologically characterized by two key parameters: proliferation rate and invasiveness. However, there are currently no standardized methods to quantify these parameters. Non-invasive imaging, such as magnetic resonance imaging (MRI), has been proposed to estimate tumor proliferation and invasiveness. Advanced mathematical models, using patient-specific radiographic data, provided significant insights into GBM behaviour. Of these, the proliferation–diffusion model is one of the most widely studied [4]. This model characterizes tumor aggressiveness through two key parameters: proliferation rate (ρ) and diffusion rate (D), derived from T1 post-gadolinium and T2/FLAIR imaging signal volumes.

The impact of the extent of surgical resection (EOR) on prognosis has been extensively studied, with recent trends in neuro-oncological surgery highlighting the value of achieving the maximum safe resection beyond the contrast-enhancing tumor boundaries. This technique, known as Supramaximal resection (SUPR) in the revised RANO criteria [4], has gained growing recognition. Building on these assumptions, our single-center retrospective study aimed to evaluate the role of the tumor invasiveness profile, quantified by the ρ/D ratio, in a well-defined cohort of patients with newly diagnosed GBM classified according to the 2021 WHO-CNS criteria that underwent SUPR as outlined by the RANO criteria, and to analyze its impact on survival outcomes.

## 2. Materials and Methods

### 2.1. Study Design and Patient Population

Patients with newly diagnosed, histologically confirmed glial tumors featuring contrast-enhancing lesions, who underwent surgical resection at our institution between January 2007 and January 2024, were retrospectively reviewed. The exclusion criteria were as follows: (1) age < 18 years, (2) prior surgical resection or biopsy, (3) presence of an IDH mutation, (4) incomplete pre- or post-operative MRI data, (5) previous radiotherapy and/or chemotherapy, and (6) patients who did not undergo SUPR resection. Demographic, clinical, histomolecular, imaging, treatment, and outcome data were collected for all included patients. This study was approved by the Humanitas Research Hospital Ethics Committee [approval n. 58/24, CET Lombardia] and conducted in compliance with the Declaration of Helsinki.

### 2.2. MRI Imaging

Preoperative volumetric MRI scans were obtained using a 3 Tesla Siemens MAGNETOM Verio MRI scanner (Siemens Medical System, Munich, Germany). The imaging protocol included T1-weighted sequences both pre- and post-gadolinium administration, as well as T2-weighted and FLAIR scans. In most cases, additional sequences such as DWI, fMRI, and DTI were also performed. Postoperative MRI was performed at 48 h and 2 months after surgery to assess the extent of resection.

### 2.3. Surgery

A neuronavigation system incorporating preoperative MRI (T2 FLAIR, T1 post-contrast) and volumetric CT scans was employed in all surgical procedures to guide craniotomy planning and define tumor margins. Preoperative volumetric T1-gadolinium-enhanced and FLAIR sequences were uploaded and merged within the BrainLab AG neuronavigation platform. Intraoperative neurophysiological monitoring included continuous electroencephalography (EEG), electrocorticography (ECoG), multichannel recordings of free-running electromyographic (EMG) activity, somatosensory-evoked potentials (SSEPs), and motor-evoked potentials (MEPs), as previously described by our group [5]. Intraoperative language and motor cortex mapping were performed when indicated. Awake surgery was conducted for selected cases involving lesions in the dominant hemisphere. Surgical resection followed functional boundaries, aiming for maximal safe resection while preserving motor, language, and visual pathways.

### 2.4. Histopathological Analysis

Histological and molecular evaluations were performed for all cases and classified following the 2021 WHO CNS tumor classification guidelines [6]. IDH status was assessed using immunohistochemical staining, with molecular testing conducted for cases with negative immunohistochemistry results. Tumor samples were fixed in buffered formalin, embedded in paraffin, and subjected to routine histologic examination. Hematoxylin-eosin (H&E)-stained slides and immunohistochemical (IHC) analyses were performed for all specimens. MGMT promoter methylation status was determined through DNA extraction from tumor tissue followed by pyrosequencing (Diatech Pharmacogenetics, MGMTplus, CE, and IVD validated). At our institution, methylation levels exceeding 5% are classified as positive, while levels ≤5% are considered negative.

### 2.5. Postoperative Treatments

Each patient was assessed by a multidisciplinary team comprises of neurosurgeons, neuro-oncologists, neuroradiologists, and radiotherapists to determine the optimal therapy. Surgery was followed by radiotherapy (RT) with concurrent temozolomide (TMZ) and subsequent maintenance TMZ, administered within 4 to 6 weeks according to the Stupp regimen [2,7,8].

### 2.6. Image Analysis

The preoperative total tumor volume (T-TV), contrast-enhancing tumor volume (CE), and infiltrative FLAIR tumor volume (FLAIR-TV) were measured in cubic centimeters (cc) through manual segmentation of contrast-enhanced T1-weighted and T2-weighted FLAIR sequences using the iPlan Cranial v3.0 software (Brainlab, Feldkirchen, Germany). Tumors without contrast enhancement on preoperative MRI were excluded. Tumor location was classified based on functional proximity to eloquent brain areas using the Sawaya et al. grading system [9]: eloquent, near-eloquent, and non-eloquent.

The early postoperative MRI was analyzed to evaluate residual contrast-enhancing and FLAIR hyperintense tumor volumes, including the calculation of residual FLAIR volume (rFL-v). To ensure accuracy, non-specific enhancements due to cerebral inflammation, blood deposition, or hemostatic agents were identified by two independent observers (M.P.T., E.B.) and excluded under the supervision of an experienced neuroradiologist (L.S.P.). Residual tumor volume (RTV) was defined as any detectable pathological abnormality on postoperative MRI performed within 48 h of surgery, corresponding to abnormalities observed in preoperative imaging.

All patients were categorized based on the 2022 RANO-Resect classification for EOR. Near-total resection (NTR) was defined as residual contrast-enhancing volume >1 cm^3^ regardless of FLAIR disease at postoperative images. Complete resection (CR) included patients with no residual CE-enhancement but a postoperative FLAIR volume >5 cm^3^. Supramaximal resection (SUPR) was defined as the absence of residual CE tumor with a postoperative FLAIR volume <5 cm^3^ [10]. Patients whose EOR did not meet the SUPR criteria were excluded from the analysis.

The tumor invasiveness profile was assessed using the proliferation/diffusion (ρ/D) ratio, calculated following Swanson et al.’s method:

ρ/D ratio = (4π/3)^2/3^ × (6.106/[VT2^1/3^ − VT1^1/3^])^2^ (1)

where VT2 represents the preoperative T2/FLAIR tumor volume, and VT1 represents the contrast-enhancing tumor volume [10,11].

**Figure 1:**
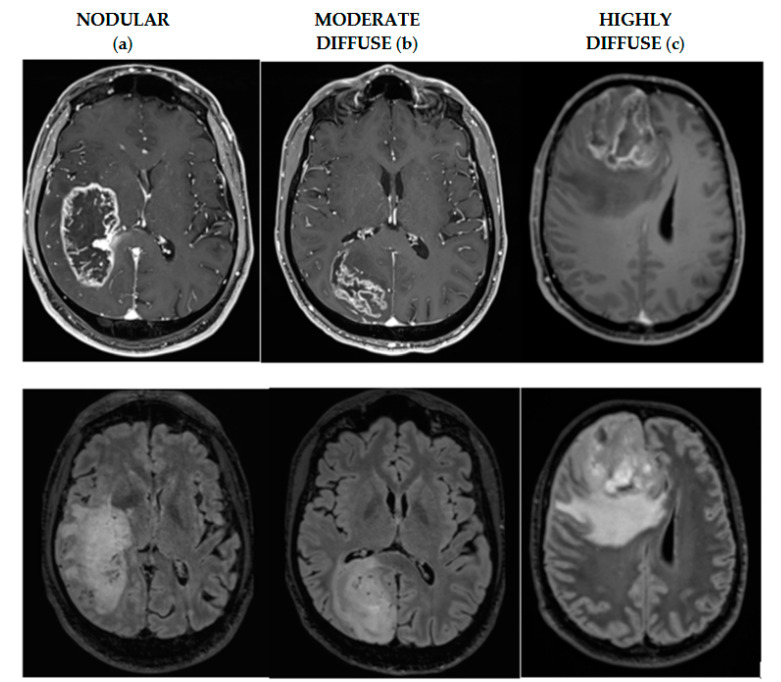
Axial MR images illustrating the classification of IDH–wild-type GBM based on the ρ/D ratio: (**a**) nodular tumor with a ρ/D ratio > 1.80 mm^−2^; (**b**) moderately diffuse tumor with a ρ/D ratio between 0.55 and 1.80 mm^−2^; (**c**) highly diffuse tumor with a ρ/D ratio < 0.55 mm^−2^.

Patients were stratified into subgroups based on tumor invasiveness: highly diffuse tumors with a low ρ/D ratio a low ρ/D ratio (<0.55 mm^−2^), moderately diffuse tumors had a moderate ρ/D ratio (0.55–1.80 mm^−2^), and nodular tumors had a high ρ/D ratio (>1.80 mm^−2^). (Figure 1).

The literature lacks consensus on standardized cutoffs for these classifications. Consequently, our analysis employed the characterization thresholds reported by Baldock et al. [10]. Additional analyses were performed using different cutoff values from other studies for comparison. Specifically, highly diffuse tumors have a low ρ/D ratio (<0.38 mm^−2^), moderately diffuse tumors have a moderate ρ/D ratio (between 0.38 and 1.30 mm^−2^), and nodular tumors have a high ρ/D ratio (>1.30 mm^−2^) [12].

### 2.7. Outcome Evaluation

Postoperative functional outcomes were evaluated through neurological examinations. The extent of resection was assessed using postoperative MRI conducted within 48 h of surgery. Follow-up neurological evaluations and MRI scans were scheduled at 3- to 6-month intervals. Disease recurrence or progression was defined based on the Response Assessment in Neuro-Oncology (RANO) criteria [10].

### 2.8. Statistical Analysis

Descriptive statistical analyses were used to summarize patient characteristics. Continuous variables were expressed as means with standard deviations or as medians with ranges, depending on the distribution’s normality, which was evaluated using the Shapiro–Wilk test. Categorical variables were reported as absolute counts and percentages.

For outcome analysis, Kaplan–Meier curves were constructed to evaluate progression-free survival (PFS) from the date of surgery to disease progression and overall survival (OS) from the date of surgery to patient death. Proportional hazards Cox regression univariable analysis was conducted to examine associations between glioblastoma outcomes and factors including gender, age, MGMT promoter methylation status, tumor location, and postoperative Karnofsky Performance Status (KPS). Variables with a *p*-value < 0.2 in univariable analysis were included in multivariable analysis for both PFS and OS. Results were expressed as hazard ratios (HRs) with 95% confidence intervals (CIs).

A significance threshold of *p* < 0.05 was applied. All statistical analyses were performed using Stata version 18 (StataCorp. 2023, Stata Statistical Software: Release 18, College Station, TX, USA: StataCorp LLC.).

## 3. Results

### 3.1. Patients Characteristics

Between 1 January 2007 and 31 January 2024, our institution treated 410 adult patients with newly diagnosed gliomas. Among them, 65 were excluded due to an IDH mutation confirmed through immunohistochemical or molecular analysis. Additionally, patients without available preoperative and postoperative MRI data or those lost to follow-up were excluded.

All included patients underwent intensity-modulated radiotherapy with concomitant temozolomide as per the Stupp protocol, followed by adjuvant temozolomide. After volumetric analyses, 57 cases were categorized into the SUPR group. A total of 52 patients with newly diagnosed, histologically confirmed IDH1 wild-type GBM who met the inclusion criteria were included in the final analysis.

Patient characteristics are summarized in Table 1. The mean age was 59.4 years (range: 21–81 years), with 33 males (63.46%) and 19 females (36.54%). No significant differences were observed in age or sex distribution. MGMT promoter methylation was identified in 36 patients (69.23%). The mean preoperative KPS was 93.2 ± 10.1, while the mean postoperative KPS was 92.64 ± 9.60.

### 3.2. Volumetric Measurements and Calculation of Tumor Invasiveness

The interobserver agreement between the first two readers was strong (κ = 0.826).

The mean preoperative contrast-enhancing (CE) tumor volume was 31.4 ± 27.9 cc (range: 0.42–115.13), while the median preoperative FLAIR tumor volume (FLAIR-TV) was 79.9 ± 51.3 cc (range: 8.13–201.34). Patients with methylated MGMT showed a slightly higher preoperative FLAIR-TV (57.4 ± 44.3 cc) compared to non-methylated cases (46.5 ± 27.5 cc), though this difference was not statistically significant (*p* = 0.709).

To evaluate the impact of surgical quality on survival benefits in relation to the tumor invasiveness profile, the series was stratified into three subgroups based on tumor invasiveness: 20 patients (38.46%) had highly diffuse tumors, 18 patients (34.61%) had moderately diffuse tumors, and 14 patients (26.93%) had nodular tumors.

### 3.3. Survival Analysis

#### 3.3.1. Overall Survival

**Figure 2:**
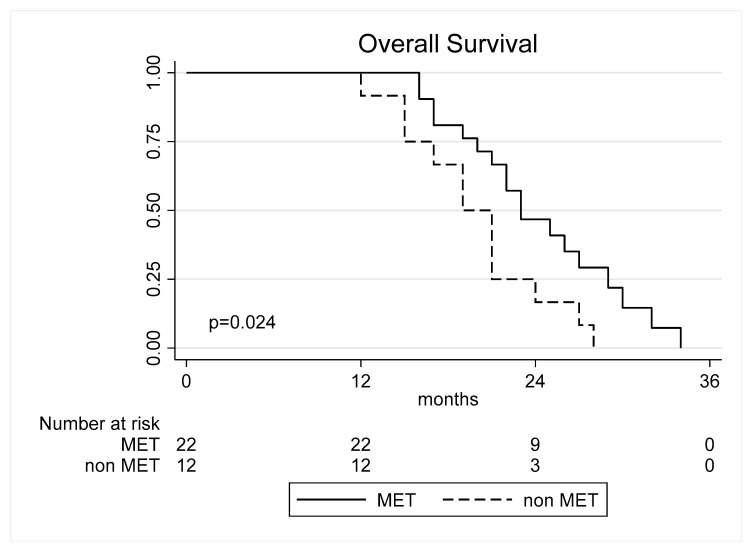
Overall survival curves according to the methylation of MGMT in moderate diffuse tumors.

The median OS was 22.88 ± 5.12 months (5–27) (Table 1). Postoperative FLAIR volume was not significantly associated with OS (*p* = 0.415). Postoperative KPS, age, and sex were not significantly associated with OS (*p* > 0.05 in all cases). Methylation of MGMT promoter was statistically significant (HR = 0.43, 95% CI: 0.20–0.94, *p* = 0.035) indicating that methylation has a protective effect on survival. No statistically significant difference was found in the subgroups according to tumor invasiveness but methylation of MGMT showed a statistically significant difference in moderate diffuse tumors (HR = 0.23; 95% CI: 0.06–0.97, *p* = 0.045, Figure 2). Following multivariate analysis, only MGMT status was confirmed to be an independent predictor of OS. These results are summarized in Table 2.

#### 3.3.2. Progression-Free Survival

The median PFS was 16.94 ± 6.18 months (12–34) (Table 1). Preoperative CE, FLAIR tumor volume, and postoperative FLAIR volume were not significantly associated with PFS (*p* = 0.158, *p* = 0.350, and *p* = 0.891, respectively). Age, sex, and pre- and postoperative KPS were not statistically significant.

**Figure 3:**
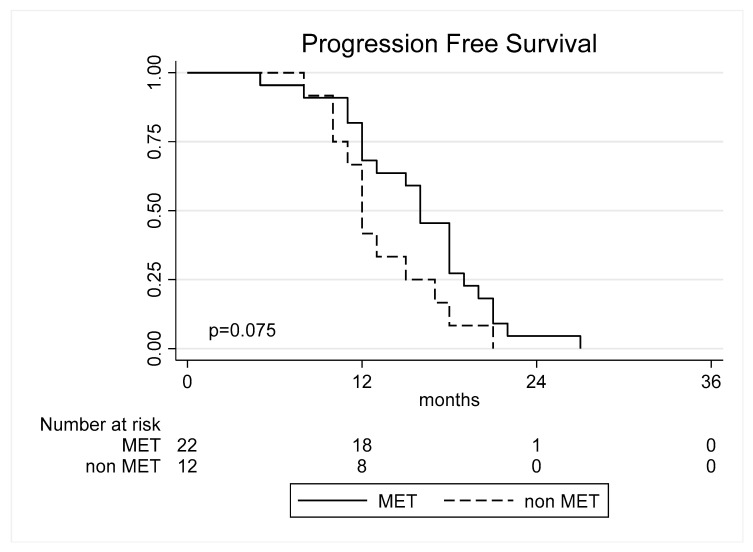
Progression-free survival curves according to the methylation of MGMT in moderate diffuse tumors.

Moderately diffuse and nodular tumors showed no significant difference in PFS compared to highly diffuse tumors (*p* = 0.821 and *p* = 0.637, respectively, Table 3). MGMT methylation was significantly associated with improved PFS in moderately diffuse tumors (HR = 0.18, 95% CI: 0.03–0.95, *p* = 0.044, Figure 3). No significant effect was observed for highly diffuse or nodular tumors (*p* = 0.888 and *p* = 0.832, respectively).

## 4. Discussion

The prognosis for patients with IDH-wildtype GBM remains poor despite recent therapeutic advancements. Even in a multidisciplinary context, surgery remains the cornerstone of treatment, and the EOR remains a topic of debate within the scientific community. Recent studies advocate for a maximal safe resection that extends beyond the contrast-enhancing boundaries of the tumor, demonstrating the superiority of extended resections (SUPR) in improving OS and PFS in GBM management [13,14,15,16,17,18]. Recently, the efforts of the RANO group have focused on establishing a standardized and unequivocal definition of SUPR emphasizing residual tumor volume rather than the amount of tissue excised during surgery [5,10].

Emerging evidence suggests that tumor invasiveness may be a predictive factor for surgical response. Among the mathematical models used to predict tumor aggressiveness, the proliferation–diffusion model is the most commonly applied [11,12]. This model evaluates tumor aggressiveness using two key parameters: proliferation rate (ρ) and diffusion rate (D), primarily derived from T1 post-gadolinium and T2/FLAIR imaging signal volumes. The product ρD determines the velocity of tumor front expansion, while the ratio ρ/D defines the extent of tumor regions that are undetectable on MRI due to insufficient cell density to produce significant changes in the imaging signal. Thus, tumors with a high value of ρ/D are more nodular, whereas tumors with a low value of ρ/D are more diffuse.

These studies naturally lead to considering whether the tumor invasiveness profile could influence the survival of patients undergoing SUPR with minimal residual FLAIR detectable. Previous studies suggest that survival benefits of the EOR differ according to the tumor invasiveness profile [11,12,13,14,15,16,17,18,19,20,21,22,23,24,25,26]. To evaluate the tumor invasiveness profile as a potential new marker for predicting GBM response to resection, we categorized tumors into three types based on the tumor invasiveness ratio (ρ/D) as reported in the literature: (1) nodular, (2) moderate diffuse, and (3) highly diffuse.

Baldock et al. [12] demonstrated that the gross total resection (GTR) provides a survival benefit for nodular tumors; however, this benefit was not observed for diffuse tumors. In contrast, Amelot et al. [11] reported that patients with diffuse and more nodular GBM experienced significant survival benefits from GTR.

Tripathi et al. [26] showed that SUPR significantly improves OS in moderate and highly diffuse IDH–wildtype GBMs with limited impact on nodular tumors. The authors suggested that in nodular tumors, GTR of the contrast-enhancing portion removes a larger proportion of the tumor burden, reducing the impact of SUPR due to lower invasiveness of the tumor cell in the FLAIR region. In contrast, moderately and highly diffuse tumors retain a greater tumor cell burden in the FLAIR region after GTR, making SUPR crucial for enhancing survival outcomes. Notably, this study defines SUPR as the removal of a variable amount of tumor beyond the contrast-enhancing area that falls within the FLAIR region, without employing the new RANO classification criteria that accounts for residual volume rather than the excised volume.

In our cohort, tumors were classified as highly diffuse in 38.46% of cases, moderately diffuse in 34.61%, and nodular in 26.93%. This study is the first to integrate the new RANO classification system for extent of resection (EOR) in glioblastoma (GBM) surgery and correlate it with the tumor invasiveness profile.

Importantly, only moderately diffuse tumors with methylated MGMT status demonstrated a statistically significant improvement in progression-free survival (PFS) (HR = 0.18, 95% CI: 0.03–0.95, *p* = 0.044) and overall survival (OS) (HR = 0.23, 95% CI: 0.06–0.97, *p* = 0.045) compared to nodular and highly diffuse tumors. In the additional analysis with the different cutoffs (highly diffuse with a low ρ/D ratio (<0.38 mm^−2^), moderately diffuse with a moderate ρ/D ratio (0.38–1.30 mm^−2^), and nodular with a high ρ/D ratio (>1.30 mm^−2^), we found the same findings.

A possible explanation for these findings lies in the interaction between tumor biology, the invasiveness profile, and the impact of surgical and adjuvant therapies. Moderately diffuse tumors, by definition, exhibit an intermediate level of cellular infiltration into the surrounding brain tissue compared to highly diffuse or nodular tumors. This intermediate profile may facilitate more effective tumor burden reduction during resection while retaining sufficient residual tumor cells responsive to adjuvant therapies such as chemoradiotherapy.

The methylation of the MGMT promoter is a well-established predictive marker of sensitivity to temozolomide [27,28,29]. In moderately diffuse tumors with methylated MGMT, the combination of reduced residual tumor burden after resection and increased chemosensitivity may synergistically contribute to the observed improvements in PFS and OS.

However, the disparity in the results reported in the literature may potentially be attributed to the lack of established cutoff values for distinguishing between nodular, moderately diffuse, and highly diffuse tumors.

A critical issue arises in utilizing the mathematical model to assess tumor invasiveness: the computation of ρ/D from MRI data relies on the assumption that the T1-Gadolinium-enhanced and FLAIR regions correspond to two distinct visibility thresholds of tumor cell density. Specifically, it is presumed that within the T1-Gadolinium-enhanced region, cell density exceeds 80% of the maximal cell density, while in the FLAIR region, it exceeds 16%.

However, this assumption may not always be true. Preoperative FLAIR imaging does not necessarily reflect tumor cells alone. Histological studies have shown that FLAIR abnormalities often correlate with edema, which may arise not only from tumor infiltration but also from vasogenic effects or inflammatory responses. These latter phenomena explain why FLAIR signal extent can be significantly reduced following surgery or steroid administration.

The lack of an MRI modality capable of accurately estimating tumor cell density within the FLAIR region represents a significant limitation to the applicability of the biophysical model.

Future studies on SUPR should focus on improving the accuracy of tumor density predictions. Integrating standard MRI sequences, such as T2-weighted imaging and diffusion-weighted imaging (DWI), with quantitative modalities like PET imaging could improve the precision of predictive models. Developing an automated, integrated system to calculate patient-specific ρ/D ratios could optimize surgical planning and standardize approaches in GBM management.

## 5. Limitations

This study has several limitations that must be acknowledged. First, its retrospective design and lack of randomization reduce the overall quality of evidence, introducing potential selection and clinical biases. The small sample size further limits the generalizability of the findings. This study focused exclusively on patients with newly diagnosed IDH1 wild-type glioblastoma to maintain cohort uniformity. Nevertheless, the potential for human error in radiographic measurements cannot be entirely ruled out. Although a rigorous methodology was applied and validated by three independent reviewers, volumetric assessments are still influenced by inherent limitations, such as differences in slice thickness and the computational constraints of the software employed.

## 6. Conclusions

Our study supports the prognostic relevance of integrating tumor invasiveness profiles with the EOR in IDH-wildtype GBM management. Using the proliferation–diffusion model to classify tumors, we identified moderately diffuse tumors with methylated MGMT status as a subgroup with significant survival benefits from SUPR. Our findings also emphasize the need for standardized definitions of tumor invasiveness cutoffs and the limitations of current imaging modalities in accurately estimating tumor cell density. Future research should focus on improving the precision of predictive models through advanced imaging techniques and automated tools for ρ/D computation, aiming to refine surgical planning and enhance survival outcomes in GBM.

## Figures and Tables

**Table 1:** Summary of descriptive analysis of the study population.

**N**	52
**Sex (M)**	33 (63.46%)
**Age (ys) (median (range))**	59.4 ± 12.3 (21–81)
**MGMT (methylated)**	36 (69.23%)
**Location**	
Near eloquent	18 (34.61%)
Eloquent	10 (19.24%)
Non-eloquent	24 (46.15%)
**Side (R)**	28 (53.84%)
**KPS (median (range))**	
Preoperative	93.2 ± 10.1
Postoperative	92.64 ± 9.60
**OS (months) (median Q1–Q3)**	22.88 (5–27)
**PFS (months) (median Q1–Q3)**	16.94 (12–34)
**Preoperative tumor volumes (cc) (median (range))**	
CE	31.4 ± 27.9 (0.42–115.13)
FLAIR-TV	79.9 ± 51.3 (8.13–201.34)
**Contact w/ventricles**	20 (38.46%)
**Tumor invasiveness**	
Highly diffuse	20 (38.46%)
Moderate diffuse	18 (34.61%)
Nodular	14 (26.93%)

**Table 2:** Univariate and multivariable Cox regression analysis of OS for the SUPR group.

Variables	Univariate Analysis		Multivariate Analysis	
	HR (95%CI)	*p*	HR (95%CI)	*p*
**Preoperative**				
CE	0.987 (0.973–1.001)	0.078		
FLAIR	0.996 (0.988–1.004)	0.368		
**Postoperative FLAIR**	1.010 (0.986–1.033)	0.415		
**MGMT (MET)**	0.43 (0.20–0.94)	**0.035**	0.392 (0.161–0.950)	**0.038**
**KPS post**	1.013 (0.970–1.057)	0.570		
**Age**	1.019 (0.983–1.055)	0.311		
**Sex (M)**	0.69 (0.32–1.48)	0.343		
**Tumor invasiveness**				
Highly diffuse	ref			
Moderate diffuse	0.68 (0.28–1.64)	0.393		
Nodular	0.90 (0.35–2.31)	0.828		
**MGMT (methylated)**				
Highly diffuse	0.80 (0.23–2.78)	0.725		
Moderate diffuse	0.23 (0.06–0.97)	**0.045**		
Nodular	0.51 (0.09–2.88)	0.444		

**Table 3:** Univariate Cox regression analysis of PFS for the SUPR group.

Variables	Univariate Analysis	
	HR (95%CI)	*p*
**Preoperative**		
CE	0.990 (0.977–1.004)	0.158
FLAIR	0.996 (0.989–1.004)	0.350
**Postoperative FLAIR**	1.002 (0.980–1.023)	0.891
**MGMT**	0.55 (0.27–1.14)	0.107
**Postoperative KPS**	0.998 (0.961–1.037)	0.936
**Age**	1.02 (0.99–1.06)	0.172
**Sex (M)**	0.94 (0.45–1.94)	0.864
**Tumor invasiveness**		
Highly diffuse	Ref	
Moderate diffuse	0.91 (0.40–2.08)	0.821
Nodular	1.22 (0.52–2.88)	0.637
**MGMT (methylated)**		
Highly diffuse	0.91 (0.27–3.09)	0.888
Moderate diffuse	0.18 (0.03–0.95)	**0.044**
Nodular	0.84 (0.16–4.28)	0.832

## Data Availability

The original contributions presented in this study are included in the article. Further inquiries can be directed to the corresponding author.
